# A Large-Scale, Cross-Sectional Investigation Into the Efficacy of Brain Training

**DOI:** 10.3389/fnhum.2019.00221

**Published:** 2019-07-09

**Authors:** Adam Hampshire, Stefano Sandrone, Peter John Hellyer

**Affiliations:** ^1^The Computational, Cognitive and Clinical Neuroimaging Laboratory, Division of Brain Sciences, Imperial College London, London, United Kingdom; ^2^Centre for Neuroimaging Sciences, King’s College London, London, United Kingdom

**Keywords:** brain training, efficacy of brain training, cross sectional study, memory, commercial brain training

## Abstract

Brain training is a large and expanding industry, and yet there is a recurrent and ongoing debate concerning its scientific basis or evidence for efficacy. Much of evidence for the efficacy of brain training within this debate is from small-scale studies that do not assess the type of “brain training,” the specificity of transfer effects, or the length of training required to achieve a generalized effect. To explore these factors, we analyze cross-sectional data from two large Internet-cohort studies (total *N* = 60,222) to determine whether cognition differs at the population level for individuals who report that they brain train on different devices, and across different timeframes, with programs in common use circa 2010–2013. Examining scores for an assessment of working-memory, reasoning and verbal abilities shows no cognitive advantages for individuals who brain train. This contrasts unfavorably with significant advantages for individuals who regularly undertake other cognitive pursuits such as computer, board and card games. However, finer grained analyses reveal a more complex relationship between brain training and cognitive performance. Specifically, individuals who have just begun to brain train start from a low cognitive baseline compared to individuals who have never engaged in brain training, whereas those who have trained for a year or more have higher working-memory and verbal scores compared to those who have just started, thus suggesting an efficacy for brain training over an *extended* period of time. The advantages in global function, working memory, and verbal memory after several months of training are plausible and of clinically relevant scale. However, this relationship is not evident for reasoning performance or self-report measures of everyday function (e.g., employment status and problems with attention). These results accord with the view that although brain training programs can produce benefits, these might extend to tasks that are operationally similar to the training regime. Furthermore, the duration of training regime required for effective enhancement of cognitive performance is longer than that applied in most previous studies.

## Introduction

Brain training is a large and expanding industry. It has been estimated that sales in this sector are increasing at a compound rate of 20 to 25% annually, passing $1.3bn worldwide in 2013 and projected to exceed $6bn by 2020 (SharpBrains, 2013; Cookson, 2014; Katz, 2014). Brain training has also been the focus of intensive academic research; however, despite the prominence and commercial success of brain training, its efficacy remains the topic of much debate.

Most notably, in 2014 more than 70 scientists signed an open letter entitled “A Consensus on the Brain Training Industry from the Scientific Community,” which argued that there is no scientific basis or evidence for the efficacy of brain training (Allaire et al., 2014). In response, 2 months later another group of more than 100 scientists publicly criticized the open letter, in form and substance, claiming that evidence for the “brain training effect” was plentiful and highlighting that the first letter could not be considered a consensus view (Alescio-Lautier et al., 2014). The latter group also accused the former of taking an extreme “faith-based” position, pertaining more to an ideological stance, whilst ignoring the scientific evidence. At present, opinions remain divided.

A number of factors contribute to this controversy. At the most fundamental level there is uncertainty regarding the definition of what exactly constitutes “efficacy”. More specifically, the core aim of brain training is to produce general improvements in cognition through repeated exercise on specific computer-based tasks. To be considered effective, brain training should enhance the performance of untrained tasks via improvements in underlying cognitive abilities (Lindenberger et al., 2017). Consequently, validation studies look for evidence of “generalization” or “transfer effects.”

Some of the largest academic randomized control trials in computerized cognitive training (ACTIVE, IHAMS and IMPACT) have reported evidence for cognitive improvement, and transfer to everyday cognitive function (e.g., IADLs/HRQoL/depression, IADLs/depression, PROs, respectively) (ACTIVE: Willis et al., 2006; Rebok et al., 2014; IHAMS: Wolinsky et al., 2013; Wolinsky et al., 2016; IMPACT: Smith et al., 2009; Zelinski et al., 2011). However, another large-scale trial published negative results in younger adults (Owen et al., 2010), although positive results, including generalization to real-world measures, were reported for older adults who trained over a longer time frame (Corbett et al., 2015). A recent meta-analyses of cognitive training and a pilot study showed benefits in cognitive function, with the first specifically noting transfer to untrained measures (Mewborn et al., 2017) and the latter reporting short-term functional and long-term structural plastic changes related to gains in global cognition (Lampit et al., 2015a, but see also Lampit et al., 2015b).

However, there is a crucial lack of clarity regarding what “transfer” actually means. In a prominent review, a differentiation between “near” and “far” transfer has been advocated (Simons et al., 2016). Specifically, “near transfer” refers to improvements that generalize to tasks that are operationally similar to the training paradigm; for example, training on one spatial working memory task and observing improvements on another spatial working memory task. In contrast, “far transfer” refers to improvements that generalize more broadly, for example, training on a spatial working memory task and observing improvements in selective attention or a composite construct such as IQ.

Indeed, to “match the hype” of the brain training sector, transfer should not only be “far”, but also ecologically valid, namely evident as improvements in everyday function. Seeking to achieve this is quite ambitious. As noted by Simons, there is “no evidence for broad-based improvement in cognition, academic achievement, professional performance, and social competencies that derive from the decontextualized practice of cognitive skills devoid of domain-specific content” (Simons et al., 2016). These broad abilities may rely on factors that brain training regime often neglect, including complex environments offering practice and engagement with domain-related challenges (Simonton, 1990; Shimamura et al., 1995; Staudinger and Baltes, 1996; Stern, 2002; Ericsson, 2006; Rohwedder and Willis, 2010; Grossmann et al., 2012). It is perhaps not surprising that only rare examples of studies reporting “far” transfer effects exist, and most of these studies used children as participants (Thorell et al., 2009; Steiner et al., 2011; Johnstone et al., 2012; Foy and Mann, 2014; Graziano and Hart, 2016; Conklin et al., 2017).

Conversely, evidence for “near transfer” is more convincing. A brain training regime was reported to improve processing speed and executive functions in the elderly (Nouchi et al., 2012) and in young adults (Nouchi et al., 2013). Substantial effects have been reported within the working memory domain for tasks that are similar to the training paradigms (Melby-Lervåg and Hulme, 2013; Karbach and Verhaeghen, 2014; Au et al., 2016; Melby-Lervåg and Hulme, 2016; Soveri et al., 2017; Strobach and Huestegge, 2017). For example, it has been shown that transfer may occur when the category of stimulus is changed and the operational requirements of the paradigm remain similar, but not when the paradigm is changed (Holmes et al., 2018). This lack of far transfer in the context of significant near transfer has also been demonstrated in a population with mild cognitive impairment (Vermeij et al., 2016). Nonetheless, some brain training studies have even failed to find even “near transfer” effects (Guye and von Bastian, 2017).

One might argue that this lack of reproducibility relates to the prevalence of too many parallel trials conducted at small cohort scale. Thus, the academic field of “brain training” has a high risk of type 1 and 2 errors. Notable exceptions to this rule are studies that have measured transfer effects in thousands of individuals. However, even there, the reported results appear contradictory, with some articles claiming significant transfer effects at large scale (Hardy et al., 2015) whereas others have reported negligible transfer even to operationally similar tasks (Owen et al., 2010).

Notably, Owen et al. (2010) have been criticized for providing insufficient “intensity” during the training phase. This criticism warrants further discussion because it highlights an often-overlooked problem: it remains unclear what the optimal parameters for a brain training regime are. Should the training last minutes or hours per session? How many times per week? What timescale should the training program be run for to produce transfer effects (near or far) of significant scale? Should brain training be paired with physical activity and social interaction to increase the positive effect of the brain training (Boot and Kramer, 2014). This issue relates to a lack of exploratory “scoping” work in the field; evidence from controlled trials forms the ultimate target of intervention research, yet this is often undertaken without prior exploration of study design parameters, which in turn inflates the risk of insensitive and underpowered studies. Brain training-wise, a gap between existing theories and existing data has very recently been highlighted (Edwards et al., 2018). While the option of dismissing effective behavioral interventions on theoretical grounds is not beneficial to public health (Edwards et al., 2018), further investigations are needed before wide implementation of brain training programs. Indeed, it is notable that older adults from the cohort of Owen et al. (2010) did show transfer effects, but they also trained over a longer period of time.

Here we attempt to bridge this knowledge gap with an exploratory cross-sectional investigation of data from two large-scale Internet-cohort studies. In the first cohort, the questionnaire included the question “do you brain train.” In the second cohort, we expanded significantly on this question in order to probe intensity, device and length of training, whilst also exploring how these factors might compare with other cognitive pursuits such as gaming. Out hypothesis was that brain training has significantly scaled transfer effects over long but not short time scales. To seek evidence of near transfer, we test whether individuals who used brain training programs in common use in 2010–2013 had a significant advantage in their working-memory, reasoning and verbal scores. We examine how these differences in scores interact with how long participants had been brain-training, i.e., for individuals who had just started to brain train compared to those who do not brain train at all and those who had trained for weeks, months or years. We then assess how cognitive performance varies as a function of training frequency. We also search for evidence of far transfer by comparing employment status and self-reported problems of attention in everyday life across the brain training groups. Finally, we test the hypothesis, that for both near and far transfer, engaging in brain training is as or more effective than alternative cognitive pursuits, including card games, video games and puzzles.

## Materials and Methods

### Cognitive Tasks

The cognitive tasks reported in this study were programmed in Adobe Flex 3 by AH. They have been reported in several previous studies (such as Owen et al., 2010; Hampshire et al., 2012; Daws and Hampshire, 2017) and were adapted for the Internet from classical paradigms in the experimental psychology and cognitive neuroscience literature. They measure planning, reasoning, attention, and working memory abilities. Tasks were presented on a bespoke web-site in a fixed sequence, after which we performed a detailed, demographic assessment. An entire battery of tasks took approximately 30 min to complete, with each task calculating one outcome measure.

### Participants

Data collection for Cohort 1 was performed between September and December 2010 via a website advertised in a New Scientist feature, on the Discovery Channel website, in the Daily Telegraph, and on social networking websites including Facebook and Twitter (for further details, please refer to Hampshire et al., 2012). Cohort 2 used a slightly different subset of tasks and was collected in the first four months of 2013 with advertisement through a press release associated with the article published from analysis of Cohort 1 (Hampshire et al., 2012).

In Cohort 1, we included participants who completed all 12 tasks in the analysis (44,780 participants, Table 1). In Cohort 2, we included in the analysis only participants who had completed 12 or more of the 13 tasks (15,442).

**TABLE 1:** Demographics for Cohort 1 (*N* = 44,780).

Age range (years)	Mean	30
	SD	11,48
Gender	Female	11,633
	Male	33,147
Handedness	Left	5,411
	Right	39,369
Brain train?	Yes	2,833
	No	41,947
Video games?	Daily	12,415
	Weekly	11,911
	Monthly	9,452
	Never	11,002

Ethical approval for the study protocol was awarded by the Cambridge Psychology Research Ethics Committees (2010.62) and the University of Western Ontario Health Sciences Research Ethics Board (103472) for Cohorts 1 and 2, respectively. All participants gave informed consent by clicking a button on the website before being able to access the cognitive and demographic assessment.

### Data Analysis

MATLAB and SPSS were used to conduct statistical analyses. The studies were not pre-registered, and the analyses are exploratory rather than resulting from an *a priori* analyses plan. Data from both studies were preprocessed using the following steps:

(i) Participants with ages below 15 or above 90 and subjects with nonsensical responses to any survey question were excluded case-wise (see Hampshire et al., 2012 for further details). Each participant was issued with a username and login. They were able to undertake the tasks multiple times if they wished; however, only their first attempt at the testing battery was analyzed in this study. Individuals who answered the questionnaire too quickly to have read the questions were excluded.
(ii) The cognitive data for each task were ranked and transformed to normality, an approach that deals with Non-normally distributed data and outlier values.
(iii) Latent variables were estimated separately from the Cohort 1 and Cohort 2 performance data in a data-driven manner using principal component analysis (PCA) as follows.

To define a “Global” measure of cognitive performance in each cohort, we first performed a principal component analysis, on the rank-transformed scores for each of the 12 tasks. The first unrotated principal component was used to define a “global” measure. Mathematically, this is the biggest linear mixture of all abilities that tasks involve and is analogous to an IQ score. To enable finer-grained analysis across different cognitive domains, we defined three orthogonal “summary” variables using a varimax rotation of the PCA coefficients. These latent variables are fully characterized in previous work (Hampshire et al., 2012; Daws and Hampshire, 2017). In brief, significant components were defined using the Kaiser convention, which only includes components that have eigenvalues that are higher than 1 (Table 2). In both datasets, three “significant” components were identified. Inspection of the task-component loadings after varimax rotation showed that these summary variables correspond to the working memory (WM), reasoning and verbal demands of the tasks. Multiple abilities underlie performance of each task, and this has been reported extensively in our previous papers. For the sake of consistency with previous studies, we used PCA with varimax rotation and not alternative methods such as PFA. We noted though, that the latter generates a near identical task-factor loading matrix.

**TABLE 2:** Brain training and computer gaming vs. task scores in Cohort 1.

		**Wald Chi-square**	***df***	**Sig.**
Global score	Video games	1413.65	3	< 0.001
	Brain training	9.98	1	0.002
WM	Video games	608.00	3	< 0.001
	Brain training	14.25	1	< 0.001
Reasoning	Video games	909.80	3	< 0.001
	Brain training	4.18	1	0.041
Verbal	Video games	18.63	3	< 0.001
	Brain training	3.10	1	0.079

(iv) Latent variable scores were generated for the participants using regression. Relationships between latent variable scores and questionnaire variables were determined by generalized linear modeling after factoring out other potentially confounding questionnaire variables.

Analyzing data with large numbers of samples affords very high statistical power, which means that effects of potentially negligible or small scale can have very low *p* values; therefore, in big-data studies of this type, a more informative gauge of significance is effect size. Here, we conform to Cohen’s notion of effect sizes, whereby an effect of ∼0.2 standard deviations (SDs) is small, ∼0.5 SDs is medium, and ∼0.8 SDs is large. All statistical values from our analyses are *p* < 0.001 unless otherwise indicated. All results and figures are presented in SD units, enabling visual assessment of effect size.

## Results

### Cohort 1 – Is Brain Training Effective?

#### Brain Training Is Effective, but the Effect Is Small to Negligible When Compared to Regular Video-Gaming

**FIGURE 1:**
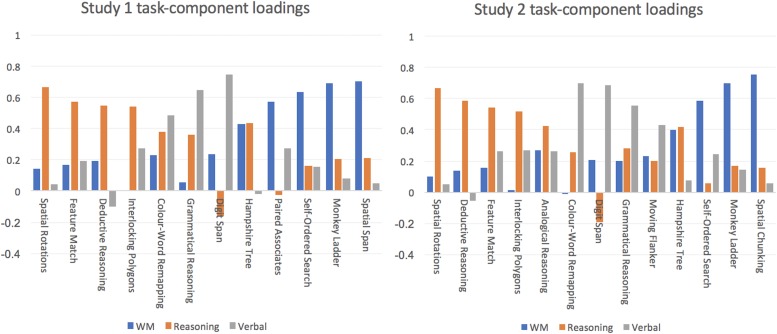
Principle components analysis. Similar varimax rotated 3-component models were evident in Study 1 and 2. One component (WM) explained substantial variance in tasks that require information to be maintained actively in working memory. Another component (Reasoning) explained variance in tasks that required either information to be transformed according to rules (e.g., Rotations and Spatial Planning) or rules to be identified (e.g., Deductive Reasoning). The final component (Verbal) explained variance in tasks that have language or number stimuli.

**FIGURE 2:**
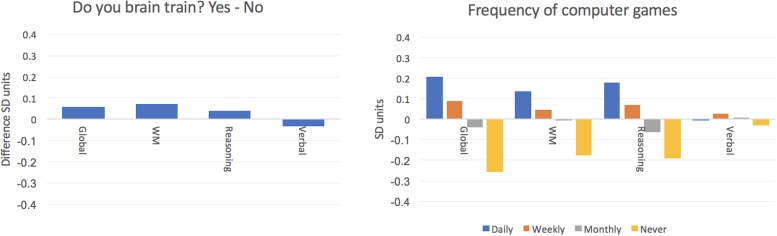
Relationship of brain training and computer gaming with cognitive score in Study 1. Left – In Study 1 there was little difference in cognitive scores for individuals who report regular brain training vs. the rest of cohort. Right – Participants who played Video Games showed small-medium scaled advantages in cognitive scores. These scaled with frequency of gaming and were evident for the Global, WM and Reasoning scores, but not for Verbal score.

Of the 44,780 participants included in Cohort 1, 2,833 reported that they regularly used a brain training program (Table 1). The global measure explained ∼28% of the population variance in performance. The three varimax rotated principal components (Figure 1), collectively accounting for ∼46% of the variance. Potentially confounding variables including age, gender, handedness, ethnicity, education level and employment status were factored out of these summary variables prior to further analysis. A general linear model was run including the factors Brain Training (answer “yes” vs. “no” to the question “Do you brain train?”) and Video Games (answer “Never,” “Monthly,” “Weekly” or “Daily” to question “How often do you play Video Games?”) with global performance as the predicted variable. The Wald Chi Squared showed statistically significant main effects of Brain Training (*X* = 9.98 *p* = 0.002) and of Video Games (*X* = 1413.7 *p* < 0.001). However, the Brain Training main effect was of small scale (+0.06 SDs). The Video Games main effect was of medium scale and there was a clear relationship with frequency of gaming, specifically, Non-gamers scored 0.47 SDs lower than those who reported playing Video Games daily. Repeating these analyses for the WM, Reasoning and Verbal summary variables (Table 2 and Figure 2) showed negligible scaled main effects for Brain Training. There were significantly scaled main effects for Video Games for the WM and Reasoning variables, but not for the Verbal variable (0.31, 0.37 and 0.024 SDs, respectively). In a final analysis, the scale of the Brain Training effect was examined separately for each age decade. None of the age groups showed a significantly scaled effect, with the largest being for people in their 30 and 60 s (both ∼0.15 SDs).

### Cohort 2 – What Are the Factors Affecting Brain Training and Other Cognitive Pursuits?

A plausible explanation for the lack of relationship between cognitive performance and brain training in Cohort 1 was that lower than average cognitive ability motivates people to engage in brain training. If this was the case, then individuals who have just started to brain train would have lower than average task performance, which would mask any benefits. A related possibility was that training may be required at high frequency produce a generalized effect. Furthermore, some training software packages may be more beneficial than others. To explore these possibilities, Cohort 2 completed a more detailed questionnaire, which included the questions “Do you believe that brain training works?,” “How often do you brain train?,” “How long have you been brain training?” and “Which brain training devices do you use?.” There also were questions pertaining to the frequency of other common cognitive pursuits including Video Games, card games, board games, and puzzles such as Sudoku and crossword puzzles.

#### Belief in Brain Training Is Consistent With Generalized Strength of Belief

**FIGURE 3:**
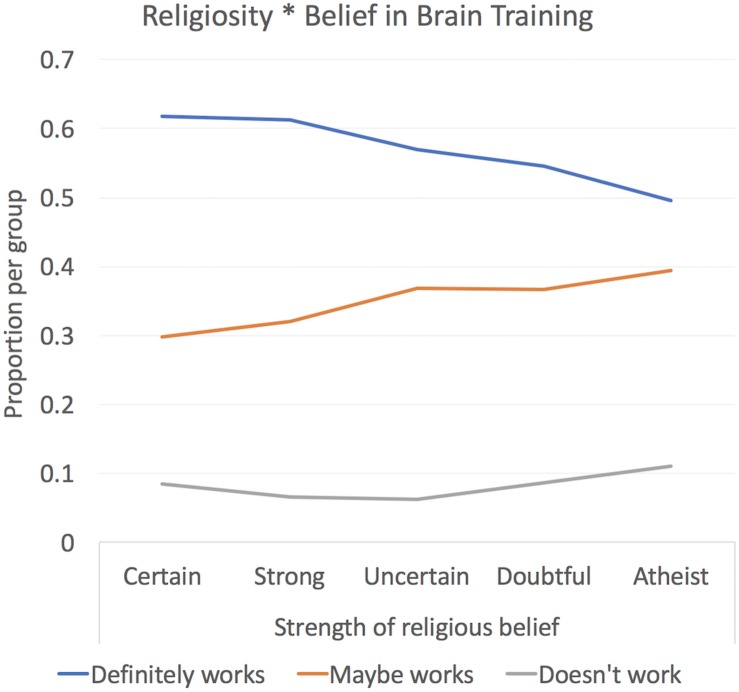
Relationship between religiosity and belief in brain training in Study 2. The majority of participants were of the opinion that brain training “works.” However, those who held strong religious beliefs also were more likely to believe in brain training.

After data cleaning, 15,442 individuals were included in Cohort 2, 3,917 of whom reported that they brain trained (Table 3). Approximately half (8,387) of the cohort answered “yes” to the question “Do you believe that brain training works,” 1,368 answered “no” with the remaining 5,682 reporting that they did not have an opinion. Interestingly, strength of belief in brain training scaled linearly with strength of religious belief (Figure 3). The global performance variable accounted for 27% of the variance in performance. The three varimax rotated components collectively accounted for 43% of the variance (Figure 1). Potentially confounding effects of age, handedness, gender, ethnicity, education level, employment status and religious group were factored out of the summary variables prior to further analysis.

**TABLE 3:** Demographics for Cohort 2 (*N* = 15,442).

Age range (years)	Mean	26
	SD	12.7
Gender	Female	4,756
	Male	10,683
Handedness	Left	1,638
	Right	13,804
Brain training works?	Yes	8,387
	Maybe	5,682
	No	1,368

#### Brain Training May Be Effective, but So Are Other Cognitive Pursuits

**FIGURE 4:**
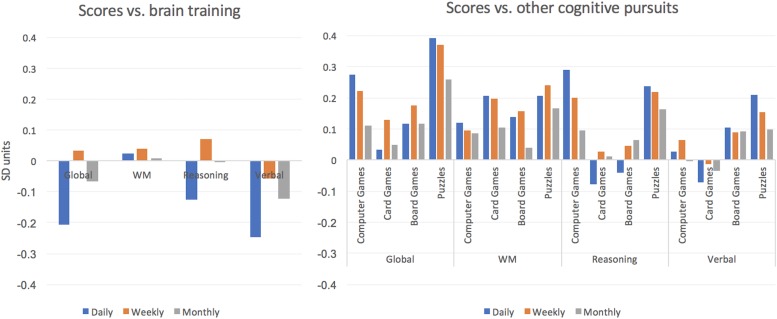
Relationship of brain training and other cognitive pursuits with cognitive scores in Study 2. Left – Cognitive scores for participants who brain train at different frequencies in cohort 2. All measures are relative to those who do not brain train. Participants who engaged in daily brain training showed a small but significant disadvantage in their Global and Verbal scores. Right – Scores broken down according to other cognitive pursuits. All values relative to participants who do not participate in the relevant cognitive pursuit. Small-medium scaled advantages in cognitive scores were evident. These often scaled with frequency. The relationships also varied according to the type of cognitive pursuit. E.g., participants who played card games regularly showed advantages for WM score only whereas those who played Video Games showed advantages for WM and Reasoning but not Verbal scores. Puzzles were associated with higher scores for all three cognitive variables.

A general linear model was run with global performance as the predicted variable and including factors for frequency (Never, Monthly, Weekly, Daily) of pursuits including Brain Training, Video Games, Board games, Cards and Puzzles (e.g., crosswords and Sudoku) (frequencies in Table 4). All factors showed statistically significant main effects at *p* < 0.001 (Table 5 and Figure 4). The largest positive effect sizes were for Video Games at 0.27 SDs and Puzzles at 0.39 SDs. Individuals who reported brain training daily showed a small disadvantage relative to those who did not (e.g., Daily training vs. Never = –0.21 SDs).

**TABLE 4:** Frequency of cognitive pursuits.

	**Daily**	**Weekly**	**Monthly**	**Never**
Brain training	810	1,055	2,052	11,519
Video games	3,572	3,666	3,684	4,515
Card games	441	1,312	6,218	7,466
Board games	265	1,071	6,110	7,991
Puzzles	1,304	4,968	6,428	2,276

**TABLE 5:** Cognitive pursuits vs. task scores in Cohort 2.

		**Wald Chi-square**	***df***	**Sig.**
Global score	Brain training	41.19	3	< 0.001
	Video games	177.29	3	< 0.001
	Card games	18.47	3	< 0.001
	Board games	51.43	3	< 0.001
	Puzzles	358.30	4	< 0.001
WM	Brain training	1.85	3	0.604
	Video games	32.78	3	< 0.001
	Card games	57.20	3	< 0.001
	Board games	24.52	3	< 0.001
	Puzzles	130.83	4	< 0.001
Reasoning	Brain training	17.78	3	< 0.001
	Video games	182.73	3	< 0.001
	Card games	4.25	3	0.235
	Board games	12.65	3	0.005
	Puzzles	127.00	4	< 0.001
Verbal	Brain training	65.48	3	< 0.001
	Video games	10.52	3	0.015
	Card games	4.72	3	0.194
	Board games	26.00	3	< 0.001
	Puzzles	68.20	4	< 0.001

At a finer grain, Video Game and puzzle players showed small advantages for the Reasoning variable (0.29 and 0.24 SDs, respectively), Cards and puzzle players showed small advantages for the WM variable (both 0.21 SDs), and puzzle players showed a small advantage for the Verbal variable (0.21 SDs) whereas individuals who brain trained showed a small disadvantage for the Verbal variable (–0.25 SDs).

#### There Are Small Differences Between Common Devices and Packages for Brain Training

When global variable scores were compared for the most common training software packages in the cohort, these being Lumosity (*N* = 877) Nintendo Brain Age (*N* = 298) vs. all others. There was no significant main effect of device (*p* = 0.537). Repeating this analysis at a finer grain showed no statistically significant main effect of device for the WM variable (*p* = 0.165). There were statistically significant main effects of device for the Reasoning and Verbal variables (*p* = 0.007 and *p* = 0.001, respectively). However, these were of negligible effect size, with Brain Age scoring 0.15 SDs higher than Lumosity for the Reasoning variable and Lumosity scoring 0.18 SDs higher than brain Age for the verbal variable.

#### Frequency and Intensity Are Independent Factors That Contribute to the Efficacy of Brain Training

Individuals who brain trained were examined at an even finer grain by dividing the population into groups according to whether they reported training for a year or more (875), months (704), weeks (695), or had just started (1644). A general linear model was run with global performance as the predicted variable and the factors Training Frequency (Daily, Weekly Monthly) and Training Duration, and the 2-way interaction of these factors (see Table 6 for cross tabulation). Both main effects were significant at *p* < 0.001. The interaction was statistically Non-significant, which is notable given the statistical power afforded at this cohort scale.

**TABLE 6:** Cross tabs for training frequency and training duration.

	**Just started**	**Weeks**	**Months**	**>1 year**	**Total**
Daily	240	130	143	298	811
Monthly	1064	316	322	350	2052
Weekly	340	249	239	227	1055
Total	1644	695	704	875	3918

#### New “Brain Trainers” Start at a Lower Baseline in Cognitive Performance

**FIGURE 5:**
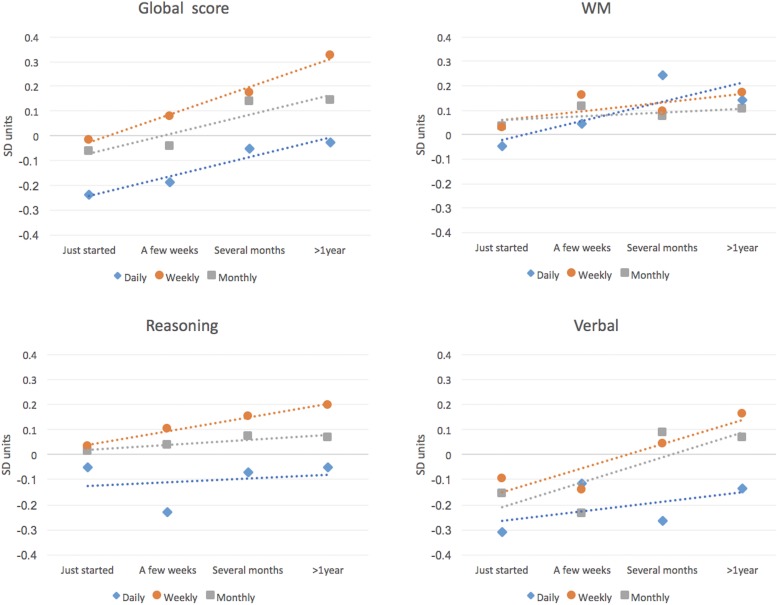
Cognitive scores in Study 2 for people who report brain training at different frequencies and over different durations. Participants who had just started brain training showed significantly scaled disadvantages in Global and Verbal score relative to participants who reported no brain training. These lower scores were most pronounced for participants who reported brain training on a daily basis. There was an increase in cognitive scores with duration of training such that those who trained weekly for a year or more had Global scores 0.32 SDs higher than the non-training population. Smaller scaled trends in the same direction were evident for the WM and Reasoning variables.

Examining the data for those individuals who had just started brain training showed that they were on average numerically below the mean performance of the broader cohort (Tables 6, 7 and Figure 5). This effect was most pronounced for those individuals who trained on a daily basis (–0.24 SDs). Global performance tracked upward in a linear manner for all three frequency groups as a function of training duration with the highest performing group being those who trained on a weekly basis for > 1 year. This group performed 0.32 SDs higher than the population average for individuals who do not brain train. Repeating this analysis for each composite performance variable showed significant main effects of frequency and duration for the Verbal variable, a significant main effect of duration for the WM variable and a significant main effect of frequency for the Reasoning variable. There were no other significant main effects or interactions (Table 7 and Figure 5).

**TABLE 7:** Main effects and interactions of frequency and duration.

		**Wald Chi-square**	***df***	**Sig.**
Global score	Frequency	28.833	2	< 0.001
	Duration	35.414	3	< 0.001
	Interaction	2.343	6	0.886
WM	Frequency	0.678	2	0.713
	Duration	12.393	3	0.006
	Interaction	5.875	6	0.437
Reasoning	Frequency	19.56	2	< 0.001
	Duration	4.461	3	0.216
	Interaction	4.232	6	0.645
Verbal	Frequency	16.739	2	< 0.001
	Duration	25.729	3	< 0.001
	Interaction	11.723	6	0.068

##### Brain training has a negligible effect on self-report of everyday problems

**FIGURE 6:**
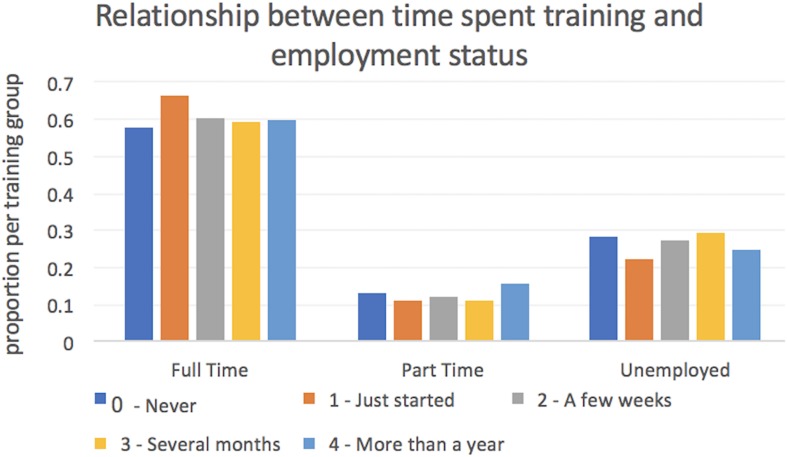
Relationship between time spent brain training and employment in Study 2. Approximately 70% of the study cohort reported being in full time employment. There was no significantly scaled relationship between employment status and the reported duration of brain training.

Finally, we examined whether the relationship that was evident between brain training duration and cognitive performance extended to every-day life, “far transfer.” First, the frequency of self-reported problems concentrating in everyday life was examined (never, infrequently, weekly, several times a week, every day, all the time) for individuals who had brain trained for different time spans. There was a statistically significant main effect of timespan (*P* < 0.001); however, although the group with lowest self-reported scores for problems concentrating were those who had brain trained the longest, the difference relative to those who just started was of negligible scale (0.072 SDs) as was the difference relative to those who do not brain train (0.13 SDs). Then, the proportion of individuals who were employed was examined as a function of time spent brain training. Calculating the strength of association between time spent training (never, just started, weekly, monthly, > 1 year) and employment status (full time, part time and unemployed) again showed a statistically significant but negligible-scaled association (Cramer’s V = 0.05, *p* < 0.001) (Figure 6).

## Discussion

Our large-scale cross-sectional analyses provide population-level insights into the likely efficacy of different brain training programs when applied at different intensities and temporal scales. The findings can help not only in the evaluation of previously reported results, but also in the design of future trials (Seitz, 2017).

At a first pass, we found little cross-sectional evidence for beneficial effects of brain training. More specifically, analysis of performance scores in Cohort 1 showed no advantage for people who brain train vs. those who do not in terms of global or any of the three summary variables. The same was essentially the case for Cohort 2; however, the extended questionnaire allowed this null finding to be examined in more detail.

The most notable finding from this finer grained analysis was that people who have just started to brain training (i.e., such that there was no time to have gained any benefits) tend to have a disadvantage relative to the broader population. Scores then track upward as a function of how long but not how frequently they have trained for. These low scores are best accounted for by motivational factors. Simply put, lower cognitive ability is likely a motivating factor for engaging in brain training. In accordance with this view, far more people believe that training works than engage in it. Furthermore, individuals who train the most frequently show the lowest starting baseline. Accounting for the differential baseline and its relationship to population variability in motivational factors is an important consideration for any future studies.

The higher performance observed in those who train for longer durations is more promising. It could well be the case that although those who engage in brain training start from a lower than average cognitive baseline, through practice they are able to improve. It is important to note that our results are from cross sectional data and must be corroborated through longitudinal trials. Nonetheless, a synergy exists between observational research and controlled trials, with the former helping to provide a guide to the focus and design of the latter, whilst the latter provides evidence for cause-effect relationships that cannot be directly inferred from the former. In the case of brain training, we argue that there has been insufficient observational research, leading to suboptimal design in many published brain training studies. Few studies have sought to determine the training timespan that is required to produce transfer effects. The guidance of observational studies can help inform optimal parameter ranges. The large-scale observational study presented here provides novel insights that may guide the design of future trials. Most notably, the significantly scaled differences in cognitive performance within the brain training group were observed when comparing those who had been training for a year or more with those who had just started. On the one side, many among the previous studies have operated at substantially shorter timescales: findings from training studies conducted at the scale of weeks or months should be treated cautiously, especially when conducted in small cohorts. On the other side, the differences between the two groups could be explained by selective attrition (e.g., people with low cognitive function are not able to sustain brain training for several months or a year), although our study is not suitable to directly assess this aspect.

Our analyses also showed no interaction or main effect of brain training frequency. On the surface, this provides little support for high intensity regimes, which, from a pragmatic perspective, is important if longer time scales are required. Although previously published reports have suggested a relationship between training frequency and the scale of transfer effects (cited in the review by Simons et al., 2016), the extent and the significance of this relationship remains elusive. *De facto*, this relationship is often assessed retrospectively, which is an aspect directly linked to the nature of the experimental study design used, and the impact of frequency on the size of the transfer may hide the contribution of other factors. A possible explanation for this can thus be the greater sensitivity and control afforded by longitudinal within-subject designs. Moreover, another explanation encompasses the fact that there may be an interaction between baseline ability and rate of improvement with training (higher ability individuals may tend to learn faster) or even an important role of other factors such as motivation. These factors may effectively cancel each other out, thus nullifying their effects. Future controlled intervention trials will assess whether intensity of training plays a key role and will clarify the relationship between training frequency and the scale of the transfer effect. Regardless, such trials should be conducted at longer duration. In line with this interpretation, the study by Corbett et al. (2015) showed cognitive and functional improvements in older adults with a brain training program over a longer timescale (Corbett et al., 2015). A possible complication with longer regime training is that dropout rates may be higher for individuals who start from lower cognitive baselines. This could conceivably produce the illusion of an improvement for groups who had been training for longer time spans. However, we consider this to be unlikely, especially taking into account that previous longitudinal studies have observed no systematic bias in compliance for high vs. low performing individuals (Hardy et al., 2015); if anything, it is the lower performing individuals who are the most motivated to engage in the training in this study as well, at least as it can be gauged by frequency, although it could still be the case that one’s perceived improvement is itself a motivating factor.

A key issue pertains to how far the benefits of brain training generalize. In their piece, Simons and colleagues suggested that benefits likely transfer to cognitive tasks that are similar to the training paradigms. However, evidence for “far” transfer to operationally distinct tasks or improvement in everyday cognitive function after brain training is lacking (Simons et al., 2016; Lindenberger et al., 2017). Our results accord well with this view. For example, there were subtle differences in the relationship between scores for the WM, Reasoning and verbal variables and training software package, which likely relates to the different composition of paradigms that are used.

More importantly, we found little evidence of far transfer effects at the population level. Specifically, when we examined two ecologically focused self-report measures: frequency of problems concentrating in everyday life and employment status. These also showed no overall relationship with brain training and correlation with training frequencies and timescale were of small scale. Again, this result accords well with Simons’ perspective on the limited scope of transfer (Simons et al., 2016). It also highlights the importance of assessing the ecological relevance of transfer effects when designing brain training studies.

More tentatively, it could be the case that to achieve generalization to everyday function in clinical populations, it will be necessary to develop training regimes that are closely targeted to the specific operational impairments that contribute to the problems they have in everyday tasks. It may also be advisable to couch such training in a more ecologically relevant format, i.e., by designing training with real-world applications (Moreau and Conway, 2014), virtual environments or augmented reality with similarities to the everyday tasks that the individual would most benefit from improving at. Also, as everyday life involves interaction with other people, ecological validity should take into account factors linked to social interactions (Engert et al., 2017; Valk et al., 2017).

The comparison of these results for brain training to other pursuits is informative in terms of simple mean differences to controls, namely those who do not brain train. Solving puzzles such as crosswords or jigsaws is of course cognitively challenging (Fissler et al., 2017) as are a vast range of other cognitive pursuits. The relationship between frequency of video games, board games and puzzles and cognitive performance were all of significant scale relative to Non-engagement. It is interesting to note that the relationships were not homogenous, i.e., different cognitive pursuits correlated with advantages in different cognitive domains. this again accords with the notion that if there are generalized benefits of engaging in such pursuits, then they likely extend in “near” as opposed to “far” manner.

We cannot rule out the possibility that these relationships have a basis in motivational factors: motivation is very different for cognitive pursuits such as computer gaming, because these are undertaken for entertainment, those who engage in such pursuits may be more motivated to do so if they are more cognitively able and perform better. However, such robust relationships warrant further attention in future studies, with a cross sectional focus extending to baseline performance differences, and further empirical work focusing on carefully controlled “game-training” trials.

Indeed, the current literature on “video-game training” is analogous to that for brain training. For example, some studies have reported significant generalized benefits (Basak et al., 2008; Boot et al., 2008; Anguera et al., 2013; Granic et al., 2014; Mayas et al., 2014; Toril et al., 2014; Green and Bavelier, 2015; Bediou et al., 2018), whereas others have no or only modest benefits (Unsworth et al., 2015; Ballesteros et al., 2017; Sala et al., 2018), with some tentative meta-analytic evidence for both near and far transfer (Wang et al., 2016; Bediou et al., 2018). Once again, the timescales required for generalized benefits is poorly defined and may underlie this inconsistency. The need for larger cohort studies and more intervention studies with more than 30 h of training has already been argued (Bediou et al., 2018), as well as the importance of considering the role of motivational effects in order to rule out alternative explanations before attributing the effect to interventions (Foroughi et al., 2016).

A final interesting point pertains to the relationship between religiosity and belief in the efficacy of brain training. It is intriguing that, as discussed above, those researchers who hold favorable opinions of brain training accused those who do not of taking a faith based position in their open letter. We have previously published analysis of religiosity and its relationship to other variables. It is somewhat ironic that it is, in fact, religious individuals, who are characterized by faith based decisions (Daws and Hampshire, 2017), those who are most likely to believe that “brain training works,” and it might be that some people are likely to believe in claims presented to them quite generally. Moreover, belief in different contexts correlates, although there might be other potential confounds (e.g., geography, age, SES). This has implications for where purveyor of brain training technology may best target their products.

Our study has many strengths, but it is important to mention the limitations that might affect the interpretation of our main findings, such as the retrospective nature of report, its Non-experimental design and potential biases inherent to self-report. Our finding that brain training has a negligible effect without long term practice is complicated by the fact that in this cross-sectional analysis participants who underwent brain-training had a heterogenous experience (i.e., focused on a range of domains). This adds noise to our findings, and potentially deflates the scale of our inference and may play a role in explaining the null results here presented, compounded by the retrospective style of the analysis, and the self-report of brain training by participants. Assessing the relationships between the content of brain training and the specific outcomes can be considered. Naturally assessing relationships between the content of brain training, and specific cognitive outcomes should be considered in future works. However, it must be noted that analysis in an interventional experimental design would require more focused parameters for duration and frequency as identified here. On a more general level, the differences between the brain training groups who have just started brain training and those that have trained for more than a year could be explained by selective attrition (e.g., people with low cognitive function are not able to sustain brain training for several months or a year).

In conclusion, we provide cross-sectional results from two large Internet-based cohorts that accord with the view that individuals who undertake commercially available brain training regimes for long timescales gain benefits that transfer in a limited way to other computerized tasks. Motivation to engage in brain training is shown to be an important confounding factor because it correlates with baseline cognitive ability. Other types of cognitive pursuit are associated with greater performance advantages in the general population and warrant further investigation with controlled trials. Future trials aimed at validating training regimes should focus on longer time-spans, carefully control for baseline ability and motivational factors, and quantify transfer to everyday function. Clinical applications of training should focus on cognitive operations that form the specific basis of patients’ impairments in order to minimize transfer distance to everyday function.

## Ethics Statement

Ethical approval for the study protocol was awarded by the Cambridge Psychology Research Ethics Committees (2010.62) and the University of Western Ontario Health Sciences Research Ethics Board (103472) for Cohorts 1 and 2, respectively. All participants gave informed consent by clicking a button on the website before being able to access the cognitive and demographic assessment.

## Author Contributions

AH and PH made substantial contributions to the conception and design of the work, as well as to the acquisition, analysis and interpretation of data for the work, drafted the work and critically revised it for important intellectual content. SS made substantial contributions to the interpretation of data for the work and critically revised the work for important intellectual content. All authors provided approval for publication of the content and agreed to be accountable for all aspects of the work in ensuring that questions related to the accuracy or integrity of any part of the work were appropriately investigated and resolved.

## Conflict of Interest Statement

The authors declare that the research was conducted in the absence of any commercial or financial relationships that could be construed as a potential conflict of interest.
